# Kienböck’s disease in a 32-year-old female: a case report

**DOI:** 10.1097/MS9.0000000000003702

**Published:** 2025-08-12

**Authors:** Humaira Siddique, Allahdad Khan, Abdul Ahad Riaz, Ali Haider Hashmi, Abdul Sattar Anjum, Mohamed Antar

**Affiliations:** a Department of Radiology, Nishtar Medical University, Multan, Pakistan; b Department of Medicine, Nishtar Medical University, Multan, Pakistan; c Department of Pathology, Nishtar II Hospital, Multan, Pakistan; d Faculty of Medicine, Tishreen University Faculty of Medicine, Latakia, Syrian Arab Republic

**Keywords:** case report, kienböck, lunate bone, osteonecrosis, wrist

## Abstract

**Introduction and Importance:**

Kienböck’s disease, or avascular necrosis of the lunate bone, is a rare condition with an estimated prevalence of 7 per 100 000 individuals. It typically affects males aged 20–40 years and often presents with wrist pain, decreased grip strength, and reduced mobility. Early diagnosis and appropriate management are crucial to prevent long-term joint destruction.

**Case presentation:**

We present a case of a 32-year-old female with chronic right wrist pain and reduced grip strength for 2 years. Examination revealed tenderness over the lunate and decreased wrist motion. Imaging confirmed sclerosis and avascular necrosis of the lunate (Stage II Lichtman classification). Conservative management with NSAIDs and physical therapy was initiated, followed by radial shortening osteotomy.

**Clinical discussion:**

Kienböck’s disease diagnosis relies on radiography, MRI, and occasionally bone scintigraphy. Early stages benefit from conservative management, while surgical intervention is recommended for more advanced cases. Radial shortening osteotomy has shown excellent outcomes in pain relief and functional recovery. Our patient showed significant improvement in pain and wrist mobility after surgery and rehabilitation.

**Conclusion:**

This case highlights the importance of early diagnosis and stage-specific management of Kienböck’s disease. Combining non-surgical and surgical approaches can achieve favourable outcomes. Longer follow-up is necessary to monitor for potential late complications.

## Introduction

Kienböck’s disease or lunatomalacia is the avascular necrosis of the lunate bone. This disorder was discovered and named after Austrian radiologist Robert Kienböck in 1910^[1–3]^. Kienböck’s disease is considered to be a rare disease and prevalence is estimated at 7 per 100,000 individuals^[4]^. Kienböck’s disease is more common in males in the age group of 20–40 years^[5,6]^. The exact cause of this disease is relatively unknown. It is believed to be caused by multiple factors including repetitive trauma, changes in blood flow, microtrauma, differences in the shape of the lunate bone, and abnormal stress on the bone^[7,8]^. Kienbock’s disease usually presents in the dominant hand^[9]^. Symptoms may include wrist pain, weakened grip strength, and reduced movement in the affected joint. Here we present a rare case report of a 32 years old female diagnosed with kienbock’s disease. This case report has been reported according to the SCARE 2025 guidelines^[10]^.

**HIGHLIGHTS**

- Kienböck’s disease is a rare cause of chronic wrist pain, often leading to functional impairment if untreated.
- Early diagnosis using radiographs and MRI is critical for appropriate staging and management.
- This case demonstrates successful management of Stage II Kienböck’s disease with radial shortening osteotomy after initial conservative treatment.
- A combined approach of medical therapy, physical rehabilitation, and surgical intervention led to significant symptomatic and functional improvement.
- Long-term follow-up is essential to monitor disease progression and prevent late complications.

## Case presentation

A 32-year-old right-handed woman of Pakistani origin presented to our outpatient clinic with a 2-year history of chronic right wrist pain and progressively declining grip strength. The pain, described as constant and dull, worsened with wrist movements such as lifting or carrying household items. She denied any prior trauma, repetitive strain, or identifiable mechanical cause. There were no systemic complaints, and no relief was reported with rest or position changes. Her occupation as a housewife did not involve high-demand manual labor. She had no significant past medical history, including diabetes or connective tissue disease, and was a non-smoker. There was no family history of musculoskeletal disorders or autoimmune diseases.

On physical examination, her left wrist exhibited full, pain-free range of motion. The right wrist, however, was tender over the lunate with mild swelling and restricted motion, especially on extension and ulnar deviation. Grip strength testing using a Jamar dynamometer revealed 12.8 kg of force in the right hand compared to 21.4 kg in the left, indicating approximately 60% strength loss. Neurological examination was normal, with no evidence of sensory deficits, and both Tinel’s and Phalen’s tests were negative, making carpal tunnel syndrome unlikely. Laboratory investigations, including serum calcium, phosphate, parathyroid hormone, and uric acid levels, were within normal limits, helping to exclude metabolic and inflammatory arthropathies.

**Figure 1.**
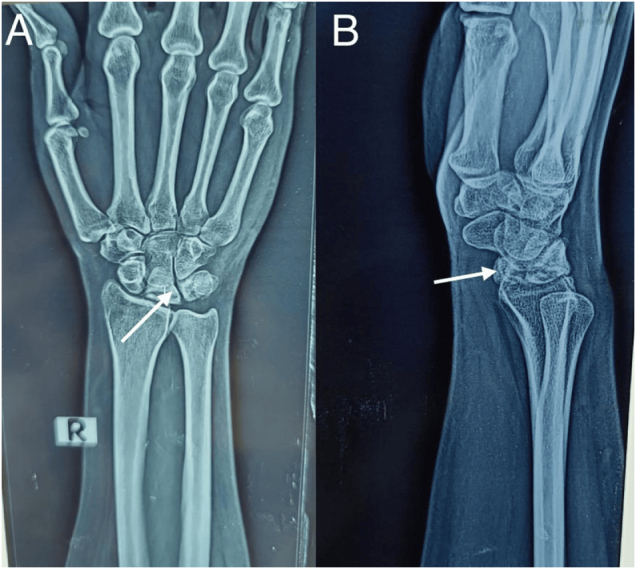
X ray right wrist, frontal view (image A) and lateral view (image B) showing sclerosis and flattening of lunate bone (arrows).

**Figure 2.**
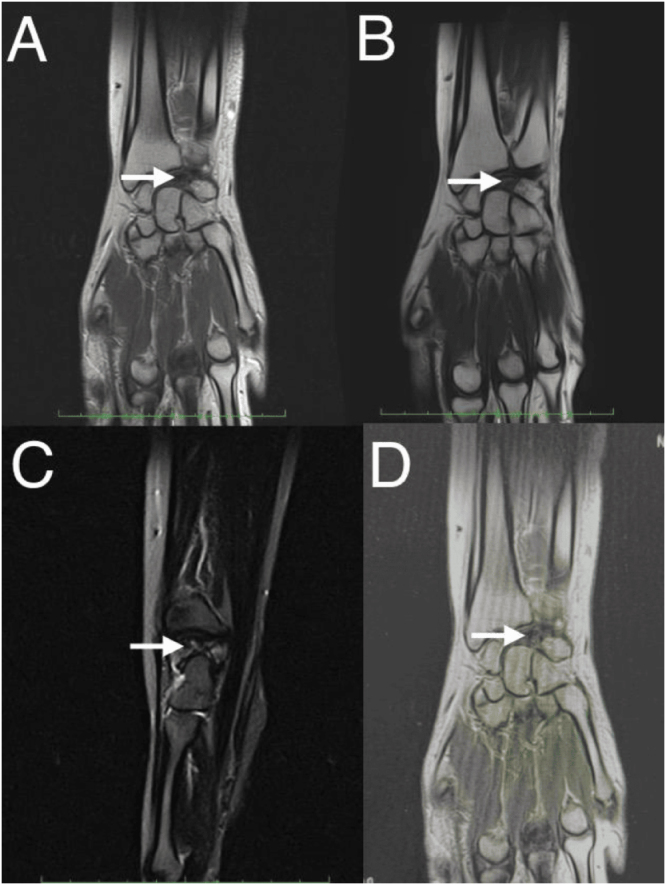
A: MRI of right wrist, coronal T1W sequence showing, relatively smaller sized lunate with hypointense signal (arrow). B: MRI of right wrist, coronal T2W sequence showing hypointense signals from lunate bone (arrow). C: MRI of right wrist, sagittal T1W sequence with fat suppression, showing hypointense signals (arrow). D: MRI of right wrist, coronal T1W sequence with gadolinium injection showing, hypointense signals without post contrast enhancement (arrow).

Radiographs (Fig. 1) of the right wrist showed sclerosis and increased radiodensity of the lunate without evidence of collapse or fragmentation, consistent with Stage II Kienböck’s disease according to the Lichtman classification. Importantly, ulnar variance was measured radiographically and found to be −2.7 mm, confirming a negative ulnar variance, a recognized predisposing anatomical factor. Bone scintigraphy using 740 MBq of 99mTc-MDP revealed focal increased uptake in the right wrist, further indicating localized pathology. MRI (Fig. 2) of the right wrist with contrast showed a small, hypointense lunate on T1W and T2W sequences, with no post-contrast enhancement, indicating avascular necrosis. Additionally, STIR hyperintense signals were seen in the proximal capitate, suggesting reactive marrow edema. These findings reinforced the diagnosis of Stage II Kienböck’s disease.

The differential diagnosis for chronic wrist pain with lunate signal abnormalities included carpal tunnel syndrome, inflammatory arthritis (such as rheumatoid arthritis or lupus), ulnar impaction syndrome, and lunate fracture. Carpal tunnel syndrome was excluded by the absence of paresthesia and negative provocative tests. Inflammatory arthritis was considered unlikely due to normal inflammatory markers, absence of systemic features, and lack of joint involvement elsewhere. Ulnar impaction syndrome was excluded due to the presence of negative, rather than positive, ulnar variance. A traumatic lunate fracture was ruled out based on clinical history and imaging that demonstrated chronic avascular changes without fracture lines.

The initial approach to management was conservative, involving NSAIDs for pain relief, wrist immobilization using a neutral splint, and referral to physiotherapy to preserve motion and strength. After 3 months of conservative treatment with minimal clinical improvement and persistent pain, a surgical option was considered. Given the presence of negative ulnar variance and the preserved lunate architecture, a radial shortening osteotomy was chosen. Given the presence of negative ulnar variance and preserved lunate architecture, a radial shortening osteotomy was selected to unload the lunate and promote revascularization. The procedure was performed under regional anesthesia with the patient in a supine position and the arm on a hand table. A standard volar approach to the distal radius was used. Intra-operatively, no synovitis or joint effusion was noted. The lunate appeared intact without signs of collapse or fragmentation, confirming preoperative imaging findings. The distal radius was marked, and a transverse osteotomy was performed approximately 10 mm proximal to the radiocarpal joint. A segment of 2.5 mm was removed to correct the negative ulnar variance.

Fixation was achieved using a 3.5 mm dynamic compression plate (Synthes), ensuring stable alignment and appropriate radial height. Intraoperative fluoroscopy confirmed satisfactory shortening and hardware placement. The total surgical time was approximately 90 minutes, with no intraoperative complications.

Postoperatively, the wrist was immobilized in a volar splint for three weeks. At the start of week 4, passive range of motion exercises were initiated under the supervision of a hand therapist. By week 7, the patient progressed to active range of motion and grip-strengthening exercises. At week twelve, dynamic splints and proprioceptive biofeedback tools were incorporated to facilitate functional recovery. Full return to non-strenuous daily activities was permitted by week 16. The surgery was performed by a fellowship-trained orthopedic hand surgeon with over 10 years of experience and a case volume exceeding 50 wrist osteotomies.

The decision-making process included discussion of alternative options such as vascularized bone grafting and proximal row carpectomy (PRC). However, the absence of lunate collapse and the relatively early disease stage made PRC less suitable, while vascularized bone grafting was deemed unnecessary given the surgical objective and anatomical context. Literature comparing these techniques suggests that radial shortening osteotomy yields favorable long-term outcomes in patients with early-stage Kienböck’s and significant negative ulnar variance.

At the 6-month follow-up, the patient reported significant pain relief and improved wrist function. Range of motion in the affected wrist was nearly restored, with extension reaching 60° and flexion 65°. Grip strength improved to 17.1 kg (approximately 80% of the unaffected hand). Functional recovery was quantified using the Disabilities of the Arm, Shoulder, and Hand (DASH) score, which improved from 48 preoperatively to 16 postoperatively, indicating marked improvement in daily function.

Postoperative rehabilitation included a structured protocol: immobilization for three weeks, passive mobilization from weeks 4 to 6, and progression to active motion and strength training by week 7. Biofeedback tools and dynamic splints were incorporated to facilitate proprioceptive retraining and grip recovery.

The patient tolerated the procedure and rehabilitation well, with no postoperative complications. She resumed light household activities and continues to be monitored for long-term outcomes. This case underscores the importance of individualized surgical planning in Kienböck’s disease, supported by anatomical and functional assessments, and highlights the role of radial shortening osteotomy as an effective intervention in Stage II disease with negative ulnar variance.

## Discussion

Kienbock’s disease, also known as osteonecrosis of the lunate, can result in long-term, severe wrist pain^[11]^. This uncommon condition has a prevalence of approximately 0.0066% and primarily affects men, particularly those aged between 20 and 40 years^[12,13]^. Usually only one hand is affected. Only in 4% of cases it is bilateral^[14]^.

The exact etiology of it is not well understood yet but many variables and causes are linked to it and impaired bone vascularization is the most commonly proposed cause^[15]^. Other factors such as Ulnar negative variance are also associated with it^[16]^. Leeuwen *et al* reported that 78% of Kienbock cases correlates with negative ulnar variance^[17]^.

Keinbock usually presents with reduced wrist motion with pain and swelling. In some cases the grip strength is also decreased as in our case^[4]^. Diagnosis of Kienbock can be challenging. The condition is characterized by osteonecrosis of the lunate and is frequently worsened by fractures, carpal misalignment, and eventual arthritic degeneration. These issues make diagnosis more complicated. Moreover the nonspecific symptoms of it also leads to delay in diagnosis^[18,19]^.

Diagnosis is made up on the basis of Radiological investigations. Lichtmann classification is used for X-ray. But in existing literature poor correlation has been described between lichtmann stage and symptoms^[12,20]^. Mirabello *et al* also describe a lack of correlation between function and Lichtman class^[21]^. Magnetic resonance imaging is considered the gold standard test for Kienbock disease. Although a 99mTc-MDP bone scan was performed early in this case, it is not considered first-line, especially in younger patients, due to its limited specificity despite high sensitivity^[22]^. Due to limitations of the Lichtmann staging system, a classification system based upon MRI has been introduced. The inter-observer reliability of the MRI classification is greater than that of the modified Lichtman classification. Moreover, MRI classification reflects carpal misalignment with higher fidelity and is more appropriate for classification into stages IIIA and IIIB^[23]^.

An important differential to exclude on imaging is ulnar impaction syndrome. It is more commonly associated with positive ulnar variance and sclerosis/signal change is at the proximal ulnar aspect of lunate.

There is no standard treatment for Kienbock. It is treated using both non-surgical and surgical modalities. Non-surgical treatment modalities being anti-inflammatory drugs, wrist immobilization with splints, and adjusting activities are used to relieve symptoms and help prevent the condition from worsening. It is done in stage 1 of the disease^[19]^. The surgical intervention is done upon the stage of Kienbock. Quantifying Ulnar variance is an essential step in management of KD. Raven *et al* and Watanbe *et al* reported that radial shortening osteotomy showed excellent pain relief^[24,25]^. Moreover, Watanbe *et al* reported that shortening osteotomy significantly increases lunate load hence shows favorable outcomes in cases of KD with negative ulnar variance^[25]^. Quezner *et al* reported that vascularized bone grafting plus radial shortening osteotomy give better results than osteotomy alone^[26]^.

In Stage II Kienböck’s disease, multiple surgical strategies are viable depending on ulnar variance and vascular viability. Vascularized bone graft (VBG) is the most frequently used method of treating stage II with neutral ulnar variance^[4]^

In a study by Daecke *et al*, pain improved in 20 out of 23 patients after vascularized pisiform transposition to a cored-out lunate for Lichtman stages II. Additionally, proximal row carpectomy (PRC) has also shown durable results in treatment of KD^[27]^.

Our patient was treated with both nonsurgical modalities like anti-inflammatory and surgical modality like shortening osteotomy because of the early stage of disease. Shortening osteotomy is considered a reliable long term treatment for early stage KD. Moreover, radial shortening osteotomy redistributes axial load away from the lunate, reducing peak pressure and improving perfusion^[25,28]^.

For advanced stages vascularized, pedicled, scaphoid graft combined with partial radioscaphoid arthrodesis or salvage procedures like total wrist arthroplasty are done^[4,29,30]^.

The prognosis of Kienbock is progressive leading to joint destruction in 3-5 years.

Prognosis depends upon factors like stage, age of patient at the time of diagnosis and negative ulnar variance^[29,31]^. Emerging tools such as bone turnover biomarkers and AI-based MRI analysis may offer improved prognostication and personalized care in the future^[32]^.

In our case the limitation exists that the follow up of the patient has been done only for 6 months. So we cannot anticipate any late future complications yet. But we plan continued assessment at 12 months and annually to monitor for late complications such as lunate collapse or radiocarpal arthritis. Moreover, in a study by Tabete *et al*, reported improvements in pain, ROM, and grip strength were maintained over the 10-year follow-up with only 33% patients showing mild osteoarthritic changes^[33]^.

In conclusion, our case highlights the importance of timely imaging with MRI, appropriate staging, and individualized treatment. However, longer follow-up and comparative evaluation with other treatment modalities such as VBG or PRC would provide a more robust perspective.

## Conclusion

Kienböck’s disease remains a challenging diagnosis due to its rarity, non-specific symptoms, and progressive nature. Early recognition and appropriate imaging, particularly with MRI, are essential for timely intervention. In this case, a multidisciplinary approach combining conservative management and radial shortening osteotomy resulted in significant symptomatic improvement and functional recovery. Although short-term outcomes were favorable, continued long-term follow-up is necessary to monitor disease progression and potential complications. This case highlights the importance of early diagnostic vigilance, stage-specific treatment planning, and tailored rehabilitation in optimizing patient outcomes for Kienböck’s disease.

## Data Availability

All the relevant data have been included in the manuscript itself.
